# Photobiomodulation versus light-emitting diode (LED) therapy in the treatment of temporomandibular disorder: study protocol for a randomized, controlled clinical trial

**DOI:** 10.1186/s13063-018-2444-7

**Published:** 2018-01-26

**Authors:** Luciana G. Langella, Paula F. C. Silva, Larissa Costa-Santos, Marcela L. L. Gonçalves, Lara J. Motta, Alessandro M. Deana, Kristianne P. S. Fernandes, Raquel A. Mesquita-Ferrari, Sandra Kalil Bussadori

**Affiliations:** Nove de Julho University, 235/249 Vergueiro Street, Liberdade, São Paulo 01504-001 Brazil

**Keywords:** Temporomandibular disorder, Photobiomodulation, Light-emitting diode (LED)

## Abstract

**Background:**

Temporomandibular disorder (TMD) is described as a subgroup of orofacial pain with a set of signs and symptoms that involve the temporomandibular joint, masticatory muscles, ears, and neck. TMD can occur unilaterally or bilaterally and approximately 70% of the population is affected with at least one sign. The disorder progresses with orofacial pain, muscle pain involving the masticatory and cervical muscles, joint noises (clicks and pops), joint block, mandibular dysfunction, and headache. The etiology can be abnormal occlusion and/or posture, trauma involving local tissues, repetitive microtrauma, parafunctional habits, and an increase in emotional stress. Studies have demonstrated that phototherapy is an efficient option for the treatment of TMD, leading to improvements in pain and orofacial function.

**Methods:**

The aim of the proposed study is to compare the effects of two sources of photobiomodulation in individuals with TMD. A randomized, controlled, double-blind, clinical trial is proposed, which will involve 80 individuals aged 18–65 years allocated to either a laser group or light-emitting diode (LED) group submitted to 12 sessions of phototherapy. The Research Diagnostic Criteria for TMDs will be used to evaluate all participants. Pain will be measured using the visual analog scale and maximum vertical mandibular movement will be determined with the aid of digital calipers.

**Discussion:**

This study compares the effects of two modalities of laser therapy on the pain and orofacial function of patients with TMD dysfunction. Photobiomodulation and LED therapy are treatment options for reducing the inflammatory process and pain as well as inducing the regeneration of the target tissue.

**Trial registration:**

ClinicalTrials.gov, NCT03257748. Registered on 8 August 2017.

**Electronic supplementary material:**

The online version of this article (doi:10.1186/s13063-018-2444-7) contains supplementary material, which is available to authorized users.

## Background

The temporomandibular joints link the lower jaw (mandible) to the temporal bone of the skull, which is immediately in front of the ear on each side of the head. The flexibility of the joints allows the mandible to move up and down as well as from side to side [1, 2].

Temporomandibular disorder (TMD) is described as a subgroup of orofacial pain with a set of signs and symptoms that involve the temporomandibular joint, masticatory muscles, ears, and neck either unilaterally or bilaterally [2–6]. The signs and symptoms include orofacial pain [2, 8], muscle pain involving the masticatory and cervical muscles, joint noises (clicks and pops), joint block, mandibular dysfunction [8–11], and headache [2, 7, 8]. The etiology can be abnormal occlusion and/or posture, trauma involving local tissues, repetitive microtrauma, parafunctional habits, and an increase in emotional stress [2, 7, 8, 10, 12]. With regard to prevalence, studies report that approximately 70% of the population can have at least one sign of TMD [13–15], but only 10% seek treatment [13, 15]. Pain is the most common symptom and can affect quality of life due to limitations regarding actions such as laughing, eating, speaking, and yawning [3, 10, 14].

Due to the pain and functional limitations inherent to TMD, phototherapy has been investigated and has proven to be an important treatment option for this disorder [1, 4, 5, 7-9, 11, 13, 16, 17]. Photobiomodulation and light-emitting diode (LED) therapy are treatment options for reducing pain and inflammatory processes as well as inducing the regeneration of the target tissue [18–20]. Both types of phototherapy have been suggested for the treatment of inflammatory joint conditions due to the absence of side effects, which are common with the use of non-steroidal anti-inflammatory drugs (NSAIDs) [20]. Studies have reported that lasers and LEDs operating with similar parameters produce equivalent effects [21, 22]. Different theories have been proposed to explain the analgesic action of phototherapy, but no consensus has been reached. Some authors report effects such as biomodulation, the capacity to stimulate cell division, analgesia, modulation of the production of β-endorphins, as well as increases in cortisol and protein synthesis [9, 16–18, 23–27].

Drugs are widely employed for the treatment of pain, but are expensive, which generates high costs in the public health realm, and have limited effectiveness as well as potentially serious side effects, especially when administered for prolonged periods of time. Thus, non-pharmacological, non-invasive resources for pain relief are of the utmost importance and have been widely investigated in the scientific literature.

### Hypothesis

Based on previous studies conducted by different research groups, the proposed study will test the hypothesis that photobiomodulation in the form of low intensity laser compared to LED decreases pain and improves temporomandibular joint function, thereby accelerating the return to activities of daily living.

### Objective

The aim of the proposed study is to analyze the effect of laser therapy and LED therapy on pain and function in patients with TMD and its association with neck pain.

### Expected results

At the end of the study, we expect to find a reduction in pain in the temporomandibular joint and temporal muscle region, and improvement in the function of the temporomandibular joint through photobiomodulation and LED therapy in patients with TMD, with a consequent improvement in their quality of life.

## Methods / design

### Study design

**Fig. 1:**
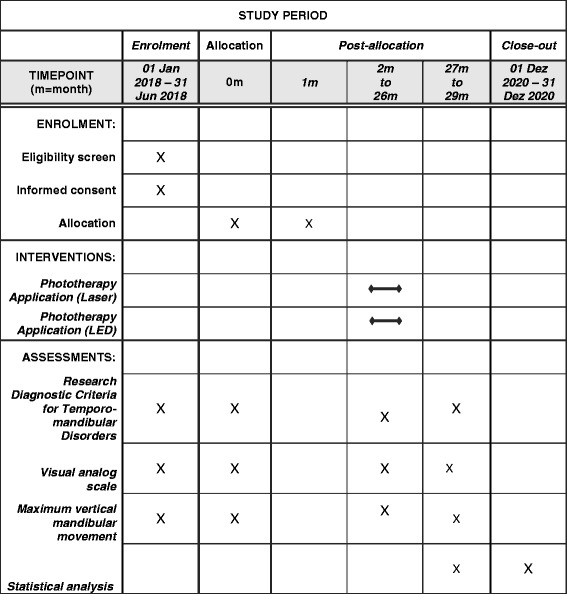
The SPIRIT Statement is important to detail the methodology, ensuring a high quality of clinical trial protocols

This protocol is presented in accordance with the 2013 SPIRIT (Standard Protocol Items: Recommendations for Interventional Trials) Statement (see Additional file 1 for the populated SPIRIT Checklist and Fig. 1 for the trial schedule of enrollment, interventions, and assessments in accordance with recommended SPIRIT figure), which was developed to provide guidance in the form of a checklist of recommended items to include in a clinical trial protocol to help improve its content and quality. A randomized, controlled, clinical trial will be conducted at the dentistry and physical therapy clinics of University Nove de Julho (Brazil) involving individuals with TMD distributed between two groups. This protocol study received approval from the Human Research Ethics Committee of University Nove de Julho (São Paulo, Brazil) under process number 1.706.160. All potential participants will receive clarifications regarding the objectives and procedures and those who agree to participate voluntarily will sign a statement of informed consent, as stipulated in Resolution 466/2012 of the Brazilian National Board of Health.

**Fig. 2:**
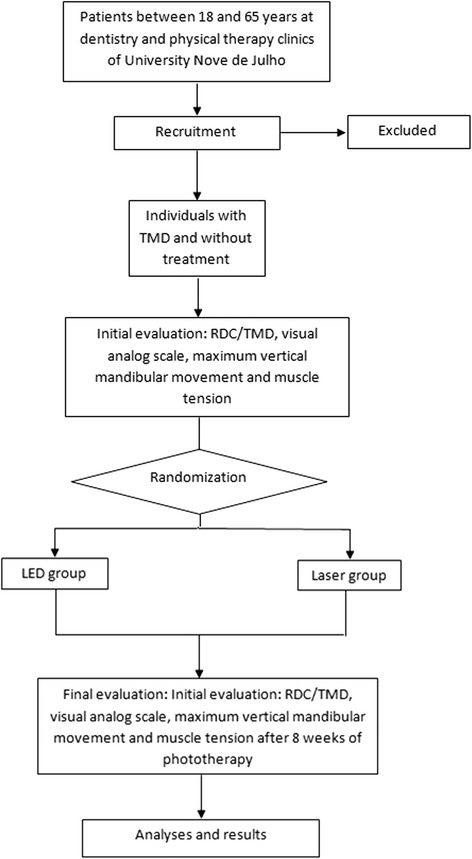
*Flow chart* illustrating phases of study (CONSORT, 2010)

The patients will be randomly allocated to either the photobiomodulation or LED group using a set of opaque, sealed, numbered envelopes to ensure concealment. Each envelop will contain a card stipulating to which group the volunteer will be allocated. The participants will then be sent for evaluations (Fig. 2).

### Participants

Male and female individuals aged 18–65 years with a clinical diagnosis of TMD based on the Research Diagnostic Criteria for Temporomandibular Disorders (RDC/TMD) will be selected to participate in the study. No restrictions will be imposed with regard to race.

The volunteers will be informed of all procedures and sign a statement of informed consent before the onset of the study.

### Inclusion criteria

- Clinical diagnosis of TMD, separated by the degrees of this function based on the Research Diagnosis Criteria for Temporomandibular Disorders, as shown in Table 1;

**Table 1:** Diagnostic subgroups according to RDC/TMD

Group I Muscle diagnoses	
Ia – myofascial pain	
Ib – myofascial pain with limited opening	
Group II Disk displacement	
IIa – disk displacement with reduction	
IIb – disk displacement without reduction with limited opening	
IIc – disk displacement without reduction without limited opening	
Group III Arthralgia, osteoarthritis, and osteoarthrosis	
IIIa – arthralgia	
IIIb – osteoarthritis of temporomandibular joint	
IIIc – osteoarthrosis of temporomandibular joint
- Age 18–65 years;
- Presence of at least 20 functional teeth, at least three being molars.

### Exclusion criteria

- Dentofacial anomalies;
- Currently in orthodontic or orthopedic treatment for the jaw;
- Currently undergoing psychological treatment;
- Use of muscle relaxant or anti-inflammatory agent.

### Sample composition

The sample size was calculated based on studies found in the literature using maximum vertical mandibular movement as the basis, with a standard deviation of 9.82, a 5% significance level (*p* < 0.05), and 80% test power. The sample was determined to be 80 individuals, who will be allocated to two groups.

- Step 1 – Recruitment for the selection of patients who will participate in the study. The patients will receive clarifications regarding the objectives and procedures and those who agree to participate voluntarily will sign a statement of informed consent.
- Step 2 – The selected patients will receive an envelope containing a card stipulating to which group they will be allocated. The patients will not be aware of the content of the envelope.

### Interventions

#### Phototherapy

The volunteers will be allocated to two groups:

The sessions will be held in a reserved, noise-free room. The volunteer will be positioned with the Frankfurt plane parallel to the ground. The devices will be covered in disposable plastic wrap to avoid cross-contamination and for hygiene reasons. The operator will wear suitable protective clothing. The skin at the site to be irradiated will have previously been cleaned with 70% alcohol. Both the operator and volunteer will use protective eyewear.

Both groups (laser and LED) will be submitted to eight sessions of phototherapy held twice a week for four weeks, during which only the researcher in charge of programming the phototherapy device will be aware of which treatment is being employed. However, the programmer will not participate in the execution of the treatments, evaluations, or data analysis.

Phototherapy will be applied:

- Temporomandibular joint region: around the temporomandibular joint;
- Masseter and temporal muscles: on the masseter and the anterior temporal.

The number of irradiated points will depend on the treatment. Patients in the laser group will have three points irradiated, while those in the LED group will have 36 points irradiated. The parameters that shall be used can be found in Table 2.

**Table 2:** Parameters of phototherapy

Parameters	Laser group	LED group
Wavelength (nm)	780	780
Target area (cm^2^)	130	130
Spot area (cm^2^)	43	3.58
Power (mW)	60	5
Irradiation time (s)	600	600
Points irradiated	3	36
Energy per point (J)	36	3
Total energy (J)	108	108
Radiant exposure (J/cm^2^)	0.8	0.8
Irradiance (mW/cm^2^)	1.38	1.38

### Outcome measures

#### Reliability of examiner

The initial evaluations will be performed by an examiner who will be duly trained by a “gold standard” evaluator following an evaluation protocol. To confer greater reliability to the results, the examiner will be blinded to the allocation of the participants to the different groups. A second examiner will perform the re-evaluations following the treatment protocols and will also be blinded to the allocation of the participants. This examiner will also be duly trained following the evaluation protocol.

### Evaluations

- Research Diagnostic Criteria for Temporomandibular Disorders (RDC/TMD): Potentially eligible patients will be diagnosed using the RDC/TMD, which consists of a clinical exam involving the palpation of the temporomandibular joints an analysis of the mandible with the aid of digital calipers to measure vertical and horizontal movements and a stethoscope to evaluate joint sounds. Symptoms such as headache, fatigue and difficulty when chewing, bruxism, psychological state, and parafunctional habits will then be investigated.
- Visual analog scale: This scale will be used for the assessment of pain and consists of a 10-cm line with 0 (absence of pain) printed at one end and 10 (debilitating pain) printed at the other end. The participants will be asked to mark a place on the line that represents their current pain intensity. The researcher will subsequently use a ruler to register the distance from zero to obtain a numeric representation of the pain level. These procedures will be performed before and after treatment.
- Maximum vertical mandibular movement: The volunteer will be instructed to open his/her mouth a wide as possible. Maximum vertical mandibular movement will be measured as the distance between the maxillary and mandibular central incisors determined with the aid of digital calipers. The volunteer will then be instructed to exert pressure on the mandibular teeth with the mouth open and move the mandible to the right and left for the determination of excursion (distance between upper and lower mid points). These procedures will be performed before and after treatment.

### Statistical analysis

The results will be tested for normality using the Kolmogorov–Smirnov test. Parametric data will be submitted to analysis of variance (ANOVA) with the Tukey–Kramer post hoc test. Non-parametric data will be submitted to Friedman’s test. The level of significance will be set to 5% (*p* < 0.05).

### Monitoring

Phototherapy has been employed for 30 years and there are no reports of side effects in the literature.

The following are the expected benefits: reductions in signs of inflammation, pain and muscle tension of the masticatory, and cervical muscles as well as an improvement in orofacial function, enabling greater mobility and functionality of the temporomandibular joints.

## Discussion

Pain and functional limitations are inherent to temporomandibular dysfunction, and studies indicate that 70% of the population is affected with at least one TMD signal [14–16]. Medications are commonly used to treat the pain, but they have a high cost and potential side effects, especially in the administration of NSAIDs [20] and long-term use [27, 28]. Therefore, there is a need for studies that evaluate the use of non-pharmacological methods for the treatment of this dysfunction.

Phototherapy has the potential to reduce pain and improve temporomandibular joint function in TMD patients, with consequent improvement in their quality of life. Photobiomodulation and LED therapy are treatment options for reduction of the inflammatory process and pain, besides inducing the regeneration of the target tissue [18–20].

The efficacy of phototherapy for the treatment of TMD has been proven by several authors [1, 4, 9, 11, 17], but there are few controlled clinical studies comparing photobiomodulation performance with LED therapy in patients with TMDs.

### Trials status

This study is currently recruiting participants.

## Additional file

Additional file 1: SPIRIT 2013 Checklist: Recommended items to address in a clinical trial protocol and related documents. (DOC 121 kb)

## Abbreviations

ANOVA: Analysis of variance
LED: Light-emitting diode
RDC/TMD: Research Diagnostic Criteria for Temporomandibular Disorders
TMD: Temporomandibular disorder
