# Correction to: Photobiomodulation versus light-emitting diode (LED) therapy in the treatment of temporomandibular disorder: study protocol for a randomized, controlled clinical trial

**DOI:** 10.1186/s13063-018-3089-2

**Published:** 2018-12-24

**Authors:** Luciana G. Langella, Paula F. C. Silva, Larissa Costa-Santos, Marcela L. L. Gonçalves, Lara J. Motta, Alessandro M. Deana, Kristianne P. S. Fernandes, Raquel A. Mesquita-Ferrari, Sandra Kalil Bussadori

**Affiliations:** Nove de Julho University, 235/249 Vergueiro Street, Liberdade, São Paulo, 01504-001 Brazil

**Correction to: Trials (2018) 19:71**

**https://doi.org/10.1186/s13063-018-2444-7**

After publication of our article [1] some questions were raised by a reader. We are grateful to the Editors-in-Chief for inviting Anne-Marie Glenny (Division of Dentistry, University of Manchester) to assess these questions, our responses and our published article. As a result we are publishing this Erratum to clarify various points. These are as follows:

- We appreciate that the title of our article does not clearly describe our protocol. Instead of “Photobiomodulation versus light-emitting diode (LED) therapy in the treatment of temporomandibular disorder: study protocol for a randomized, controlled clinical trial” a more appropriate title would be: “Photobiomodulation with laser versus photobiomodulation with light-emitting diode (LED) in the treatment of temporomandibular disorder: study protocol for a randomized, controlled clinical trial”;
- We would like to add that the sequence generation to randomize patients will be computer generated;
- We would like to make it clear that our primary outcome is pain, evaluated with a visual analog scale (VAS).
